# Chronic pain and pain-related disability across psychiatric disorders in a clinical adolescent sample

**DOI:** 10.1186/1471-244X-13-272

**Published:** 2013-10-21

**Authors:** Wenche L Mangerud, Ottar Bjerkeset, Stian Lydersen, Marit S Indredavik

**Affiliations:** 1 Regional Centre for Child and Youth Mental Health and Child Welfare, Faculty of Medicine, Norwegian University of Science and Technology. Medical Technical Research Centre, Postbox 8905, N-7491 Trondheim, Norway; 2 Department of Neuroscience, Faculty of Medicine, Norwegian University of Science and Technology, Trondheim, Norway; 3 Department of Research and Development, Levanger Hospital, Nord-Trøndelag Health Trust, Levanger, Norway; 4 Department of Child and Adolescent Psychiatry, St. Olav’s University Hospital, Trondheim, Norway

**Keywords:** Chronic pain, Disability, Prevalence, Psychiatric disorders, Adolescents

## Abstract

**Background:**

People who suffer from psychiatric disorders are burdened with a high prevalence of chronic illnesses and pain, but evidence on pain prevalence among adolescents with psychiatric disorders is scarce. The aim of this study was to investigate the frequency and location of self-reported chronic pain and pain-related disability in adolescent psychiatric patients.

**Methods:**

This study was part of the larger Health Survey administered at the Department of Child and Adolescent Psychiatry (CAP) at St. Olav’s University Hospital, in Trondheim, Norway. All patients aged 13–18 years who visited the CAP clinic at least once between February 15, 2009 and February 15, 2011 were invited to participate. A total of 717 (43.5% of eligible/invited patients) participated; of these, 566 were diagnosed with one or more psychiatric disorders. The adolescents completed a questionnaire, which included questions about pain and pain-related disability. Clinical diagnoses were classified by a clinician according to International Statistical Classification of Diseases and Related Health Problems, 10th revision criteria.

**Results:**

In adolescents with psychiatric disorders, 70.4% reported chronic pain, and 37.3% experienced chronic pain in three or more locations (multisite pain). Chronic musculoskeletal pain was the most prevalent type of pain (57.7%). Pain-related disability was found in 22.2% of the sample. The frequency of chronic pain and multisite pain increased with age, and girls reported a higher frequency of chronic pain, multisite pain and pain-related disability than boys did. There was an increased risk of chronic pain among adolescents with mood or anxiety disorders versus those with hyperkinetic disorders, yet this was not present after adjusting for sex. Comorbidity between hyperkinetic *and* mood or anxiety disorders involved an increased risk of pain-related disability.

**Conclusions:**

In this study, seven out of 10 adolescents with psychiatric disorders reported chronic pain. These findings indicate the importance of early detection of chronic pain in adolescents with psychiatric disorders, to provide targeted treatment and reduce poor long-term outcomes.

## Background

In Norway, approximately 8% of children and adolescents between 3 and 18 years meet the criteria for one or more psychiatric disorders that require treatment [1]. Starting from 12 years of age, females represent two-thirds of the adolescents diagnosed with a psychiatric disorder, and a similar ratio is seen in those who receive psychiatric treatment. Disorders such as anxiety, depression, and eating disorders are more common among girls than among boys, while hyperkinetic and conduct disorders are more common among boys than among girls [1]. Furthermore, it is well known that people who suffer from psychiatric disorders are burdened with a high prevalence of chronic illnesses and pain [2-4]. In two literature reviews, the authors found that about 50 – 65% of depressed adult patients reported pain [3,5], but evidence on pain prevalence among adolescents with psychiatric disorders is scarce. In the general population, chronic pain affects about 18% of children and adolescents (6–15 years of age) in Norway [6]. Pain prevalence rates are generally higher in females than in males and increases with age [7-10]. Furthermore, pain prevalence has been linked with low socioeconomic status (SES), but the findings are conflicting [7].

There is evidence that psychiatric disorders may increase pain intensity through a central pain modulation system, where the physiological bases of depression and pain share some of the pathways in the central nervous system [3]. Anxious patients may also interpret pain as being more intense than do non-anxious patients [11]. The presence of chronic pain makes it difficult to recognize and treat potential psychiatric disorders, and therefore this delay may worsen the prognosis of psychiatric disorders [3].

Psychiatric disorders and chronic pain, both separately and as comorbid conditions, impair the daily lives of those affected. In a large Norwegian population-based study, approximately 80% of the adolescents who reported pain in any location also reported disability in daily functioning [12]. Adolescents who experience pain often or who have mental health problems have lower school attendance rates and may have reduced academic performance compared with those without these conditions [13,14].

To our knowledge, no previous studies have examined pain prevalence, pain location and perceived pain-related disability across different psychiatric disorders in adolescence.

We investigated the frequency and location of self-reported chronic pain and pain-related disability among adolescent psychiatric patients referred to a child and adolescent psychiatric clinic in the county of Sør-Trøndelag, Norway. We hypothesized that adolescents with a psychiatric disorder would have a high frequency of chronic pain, multisite pain and pain-related disability. We also predicted that the frequency would increase with age and that girls would report a higher frequency of chronic pain, multisite pain, and pain-related disability than boys would. Furthermore, we expected to find a higher risk of chronic pain and pain-related disability for adolescents with mood or anxiety disorders than for those with hyperkinetic disorders, and an even higher risk of chronic pain and pain-related disability among those with a combination of hyperkinetic *and* mood or anxiety disorders.

## Methods

### Study setting and participants

The present study was part of the larger Health Survey undertaken at the Department of Child and Adolescent Psychiatry (CAP) at St. Olav’s University Hospital in Trondheim, Norway. It was a cross-sectional study of all patients aged 13–18 years who visited the CAP clinic at least once between February 15, 2009 and February 15, 2011. Emergency patients were also invited to participate after they were stabilized. The exclusion criteria were: considerable difficulties completing the questionnaire because of inadequate language skills, poor cognitive function or a severe psychiatric state that could not be stabilized sufficiently.

During the study period, 2032 adolescent patients visited the CAP clinic at least once. Of these, 95 were missing registration data, and hence, were not included in study recruitment. Another 289 patients were excluded according to the exclusion criteria. As a result, 1648 patients (81.1%) were eligible and were invited to participate. Of these, a total of 717 (43.5%) participated in the CAP survey. To explore the representativeness of the study population, anonymous information about the total clinical population was collected from annual reports from St. Olav’s University Hospital, 2009–2011. All adolescents in the study period (n = 2032), minus those excluded (n = 289) were defined as the reference population (n = 1743). In accordance with the permissions given by the Norwegian Social Science Data Services; The Data Protection Official for Research, we compared age, sex and main reason for referral between participants (n = 717) and non-participants (n = 1026) of our reference population. Participants were 0.27 (95% CI: 0.10–0.45) years older than the non-participants (mean (SD): 15.66 (1.65) vs 15.39 (1.95), *P* = 0.0015). There were more girls in the study group than in the non-participating group (393 (54.8%) vs. 509 (49.6%), *P* = 0.032). The main reason for referral did not differ between participants and non-participants (data not shown, exact Pearson chi-square test; *P* = 0.11).

Of the 717 participants who answered the CAP survey, 151 were not assessed fully within the study period or did not meet the criteria for a psychiatric disorder, hence the present study included 566 adolescents, who all met the criteria for at least one psychiatric disorder: 307 girls (54.2%) and 259 boys (45.8%). The mean age was 15.66 years, with 227 (40.1%) aged 13–14 years, 200 (35.3%) aged 15–16 years, and 139 (24.6%) aged 17–18 years.

Of the 566 adolescents, 24 received inpatient or ambulatory treatment, the rest were outpatients. Because of the small number of inpatients, we did not distinguish between inpatients and outpatients in our analyses.

### Procedures

Newly referred patients and patients already enrolled at the CAP clinic received verbal and written invitations during their first visit after the project started. Parental consent was obtained for participants under 16 years of age while participants aged 16 years or more gave written informed consent to participate. Parents were invited to provide supplementary information and they also gave written informed consent to participate. The participants responded to an electronic questionnaire, accessed via a password-protected website. The questionnaire was completed at the clinic, without the presence of their parents. A project coordinator provided assistance if needed. The parents responded to a shorter questionnaire, either electronically or on paper. Data from the participants were collected from their medical records.

### Measures

#### Medical records

The diagnoses were determined according to the International Statistical Classification of Diseases and Related Health Problems, 10th revision (ICD-10) multi – axial diagnostics (axes I – IV) [15]. The disorder causing the present referral, and hence, the one requiring the most treatment resources was set as the main diagnosis. Secondary diagnoses were determined according to the same criteria as the primary diagnosis. Diagnoses were made during ordinary clinical practice by a child psychiatrist or psychologist after reaching a consensus with other professionals from the multi-disciplinary team. Diagnoses were registered in the medical records, and subsequently used in this study. The CAP clinic follows standardized procedures for the assessment and diagnosis of common adolescent psychiatric disorders, including hyperkinetic disorders, autism spectrum disorders, tic disorders, psychosis, depression and eating disorders. The procedures typically require a thorough developmental history, interviews with the adolescents and parents, and the use of rating scales suitable for the presenting problem. The assessment may be supplemented with somatic examination, and possible coexisting disorders are explored. Psychological or educational testing is needed to assess learning disorders.

In this study, we classified the patients according to the main Axis I psychiatric diagnoses (ICD-10 codes are specified in Table 1): mood disorders (n = 87, of these 74 had a depressive disorder), anxiety disorders (n = 148), eating disorders (n = 22), autism spectrum disorders (n = 39), hyperkinetic disorders (n = 216) and other disorders (n = 54, of these 10 had conduct disorders, and the rest represented a broad spectrum of psychiatric disorders with low frequency).

**Table 1:** Frequency of chronic pain, pain location, and pain-related disability across psychiatric disorders in 566 adolescent psychiatric patients

	**Total sample**	**Mood (affective) disorders**^**a**^	**Anxiety disorders**^**b**^	**Eating disorders**^**c**^	**Autism spectrum disorders**^**d**^	**Hyperkinetic disorders**^**e**^	**Other disorders**^**f**^
	**n = 566**	**n = 87**	**n = 148**	**n = 22**	**n = 39**	**n = 216**	**n = 54**
**Age (years): mean (SD) n = 566**	15.7 (1.7)	16.4 (1.6)	15.8 (1.7)	16.3(1.1)	15.3 (1.5)	15.4 (1.7)	15.3(1.7)
**Chronic pain: n (%) n = 560**	393/560 (70.2)	67/85 (78.8)	113/148 (76.4)	16/22 (72.7)	22/37 (59.5)	141/216 (65.9)	34/54 (63.0)
**- Headache/Migraine: n (%) n = 551**	226/551 (41.0)	48/84 (57.1)	72/144 (50.0)	9/22 (40.9)	9/36 (25.0)	70/216 (33.2)	18/54 (33.3)
**- Musculoskeletal pain: n (%) n = 560**	323/560 (57.7)	56/85 (65.9)	95/148 (64.2)	9/22 (40.9)	18/37 (48.6)	118/216 (55.1)	27/54 (50.0)
**- Abdominal pain: n (%) n = 551**	187/551 (33.9)	38/84 (45.2)	61/144 (42.4)	12/22 (54.5)	8/36 (22.2)	50/216 (23.7)	18/54 (33.3)
**Multisite pain: n (%) n = 560**	209/560 (37.3)	45/85 (52.9)	64/148 (43.2)	8/22 (36.4)	8/37 (21.6)	67/216 (31.3)	17/54 (31.5)
**Subjective Disability Index: n (%) n = 564**	125/564 (22.2)	32/85 (37.6)	36/148 (24.3)	3/22 (13.6)	4/39 (10.3)	45/216 (20.8)	5/54 (9.3)

The numbers in this table, for example 67/85 (78.8), indicate that 67 of 85 with mood (affective) disorders had chronic pain, which shows that we had two missing values (n = 87). This applies to the entire table.

^a^F31–F34, F38–F39.

^b^F40–F 44, F48 and F93.

^c^F50.

^d^F84.

^e^F90.

^f^F20–F21, F28–F29, F54, F59–F60, F91–F92, F94–F95 and F98.

Owing to the presence of secondary diagnoses indicating comorbidity of mood or anxiety disorders with hyperkinetic disorders, we further compared three groups of patients: 1) patients with mood or anxiety disorders, but not hyperkinetic disorders (denoted MA-group, n = 223), 2) patients with hyperkinetic disorders, but not mood or anxiety disorders (denoted H-group, n = 188), and 3) patients with hyperkinetic disorders *and* mood or anxiety disorders (denoted HMA-group, n = 40).

#### Pain

The questionnaire in the CAP survey included questions about pain experienced during the last three months, not related to any known disease or injury [16]. Adolescents were asked to specify if they had experienced headaches or migraines, abdominal pain, or musculoskeletal pain (e.g. pain in the neck, shoulder, upper back, lower back/buttocks, chest, or upper and lower extremities). There was an illustration of an anatomical figure adjacent to these questions, showing the location of the named areas. The frequency of pain in each location was specified as; never/seldom (1), once a month (2), once a week (3), more than once a week (4), or almost every day (5). Chronic pain was defined as pain not related to any known disease or injury, occurring at least once a week in the last 3 months. Chronic musculoskeletal pain was defined as having chronic pain in at least one of the musculoskeletal locations (neck or shoulder, upper back, lower back or buttocks, chest, upper and/or lower extremities). Multisite pain was defined as having chronic pain in three locations or more.

#### Subjective disability index

A subjective disability index was used to measure the impact of chronic pain on daily functioning [12,17]. The adolescents answered yes or no to five statements: “I have difficulties falling asleep because of pain and aching”, “Pain or aching disturbs my (good) sleep”, “Because of pain I have difficulties sitting during a lesson”, “Pain makes it difficult for me to walk more than 1 km”, and “Pain and aching hinder me in my leisure activities”. One point was given for each affirmative answer, giving a total score of 0–5, where 0 is equivalent to pain not affecting daily living and 5 is equivalent to pain affecting all the specified areas in daily living. In this study, scores ranging from 3–5 were used to indicate disability.

#### Socioeconomic status

The highest reported educational level of the parents was used to represent SES of the adolescents. Parental education level was measured on a four-point scale: less than compulsory school or 1–2 years in high school (0–11 years), n = 53 (10.4%); completed high school, including those who had one year of education or training after high school (12–13 years), n = 226 (44.5%); academy/university for up to four years (14–15 years), n = 145 (28.5%); and academy/university for five years or more, or a PhD (16 years or more), n = 84, (16.5%), yielding a total of n = 508 (89.8%).

### Statistics

Differences in proportions were analyzed using the Pearson chi-square test. Trends in proportions were analyzed using the Cochran-Armitage test. Risk of pain or pain-related disability for adolescents in the MA-group and HMA-group versus those in the H-group was analyzed using logistic regression. Ninety-five percent confidence intervals (CI) are reported where relevant. Two-sided *P* values of < 0.05 were considered statistically significant. Data were analyzed using SPSS 19 (IBM, Chicago, USA).

### Ethics

Written informed consent was obtained from adolescents and parents prior to inclusion, in accordance with the study procedures in the CAP survey. Study approval was given by the Regional Committees for Medical and Health Research Ethics (reference numbers for CAP survey: 4.2008.1393, present study: 2011//2061/REK midt), and by the Norwegian Social Science Data Services (reference number for CAP survey: 19976).

## Results

### Frequency of chronic pain

Among adolescents who had a psychiatric disorder (n = 566), chronic pain was found in 70%, and those with mood and anxiety disorders had the highest prevalence, with 79% and 76% reporting chronic pain, respectively (see Table 1). This pattern was consistent for headache/migraine, musculoskeletal pain, multisite pain and pain-related disability. Chronic musculoskeletal pain was the most frequent type of pain (location); it was reported by 58% of the adolescents in the total sample. Specifically, 66% of adolescents with mood disorders and 64% with anxiety disorders reported chronic musculoskeletal pain. Adolescents with eating disorders had the highest prevalence of abdominal pain (55%), and the same group also had a high prevalence of chronic pain (73%). Among those with hyperkinetic disorders, chronic pain (66%) and musculoskeletal pain (55%) were the most common pain conditions.

**Figure 1:**
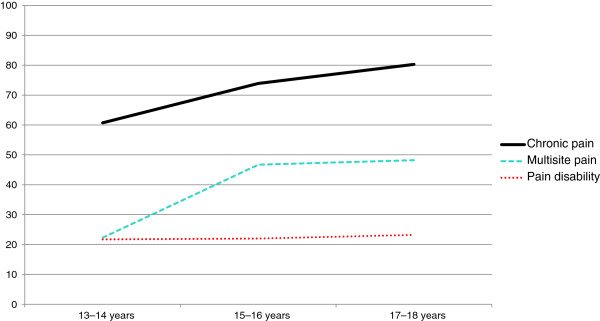
Prevalence of chronic pain, multisite pain and pain disability as a function of age in 566 adolescents with a psychiatric disorder.

Figure 1 illustrates that as participant age increased, chronic pain (OR = 1.26, CI 1.13–1.40, *P* < 0.001), and multisite pain (OR = 1.37, CI 1.24–1.51, *P* = 0.016) also increased significantly in adolescents with a psychiatric disorder, whereas pain-related disability did not (OR = 1.01, CI 0.91–1.13, *P* = 0.793).

### Chronic pain and pain-related disability in the main groups

Table 2 compares the chronic pain and pain-related disability reported by each group. Adolescents in the MA-group had a higher prevalence of chronic pain (all three locations), multisite pain and pain-related disability than those in the H-group (*P* < 0.05). In addition, adolescents in the HMA-group had a higher prevalence of chronic pain (all three locations), multisite pain and pain-related disability than those in the H-group, with statistically significant differences in chronic headaches (51.3% vs 30.6%, *P* = 0.013), chronic abdominal pain (47.4% vs 19.6%, *P* < 0.001), multisite pain (57.5% vs 26.9%, *P* < 0.001) and pain-related disability (35.0% vs 18.6%, *P* = 0.022). The HMA-group had a non-significant higher frequency of almost all types of chronic pain and pain-related disability than the MA-group did.

**Table 2:** Frequency of pain, multisite pain, and pain disability in 566 adolescents in the total sample, and in 451 adolescents in the main groups: MA-group, H-group and HMA-group, specified for girls and boys

	**Total sample**			**H-group**			**MA-group**			**HMA-group**		
	**Total**	**Girls**	**Boys**	**Total**	**Girls**	**Boys**	**Total**	**Girls**	**Boys**	**Total**	**Girls**	**Boys**
	**n = 566**	**n = 307**	**n = 259**	**n = 188**	**n = 75**	**n = 113**	**n = 223**	**n = 158**	**n = 65**	**n = 40**	**n = 25**	**n = 15**
	**n (%)**	**n (%)**	**n (%)**	**n (%)**	**n (%)**	**n (%)**	**n (%)**	**n (%)**	**n (%)**	**n (%)**	**n (%)**	**n (%)**
**Chronic pain: n = 560**	393/560 (70.4)	253/306 (82.7)	140/254 (55.1)	120/186 (64.5)	59/75 (78.8)	61/111 (55.0)	170/221 (76.9)	133/158 (84.2)	37/63 (58.7)	31/40 (77.5)	22/25 (88.0)	9/15 (60.0)
**- Headache/Migraine: n = 551**	226/551 (41.0)	169/301 (56.1)	57/250 (22.8)	56/183 (30.6)	32/74 (43.2)	24/109 (22.0)	114/217 (52.5)	97/154 (63.0)	17/63 (27.0)	20/39 (51.3)	17/25 (68.0)	3/14 (21.4)
**- Musculoskeletal pain: n = 560**	323/560 (57.7)	209/306 (68.3)	114/254 (44.9)	99/186 (53.2)	51/75 (68.0)	48/111 (43.2)	143/221 (64.7)	112/158 (70.9)	31/63 (49.2)	27/40 (67.5)	21/25 (84.0)	6/15 (40.0)
**- Abdominal pain: n = 551**	187/551 (33.9)	147/302 (48.7)	40/249 (16.1)	36/184 (19.6)	25/75 (33.3)	11/109 (10.1)	95/217 (43.8)	84/154 (54.5)	11/63 (17.5)	18/38 (47.4)	15/25 (60.0)	3/13 (23.1)
**Multisite pain: n = 560**	209/560 (37.3)	158/306 (51.6)	51/254 (20.1)	50/186 (26.9)	31/75 (41.3)	19/111 (17.1)	103/221 (46.6)	89/158 (56.3)	14/63 (22.2)	23/40 (57.5)	20/25 (80.0)	3/15 (20.0)
**Subjective Disability Index: n = 564**	125/564 (22.2)	90/307 (29.3)	35/257 (13.6)	35/188 (18.6)	21/75 (28.0)	14/113 (12.4)	64/221 (29.0)	53/158 (33.5)	11/63 (17.5)	14/40 (35.0)	9/25 (36.0)	5/15 (33.3)

H-group represents those with hyperkinetic disorders; MA-group represents mood or anxiety disorders; HMA-group represents hyperkinetic *and* mood or anxiety disorders. The diagnostic groups of “other disorders” (n = 54), “eating disorders” (n = 22), and “autism spectrum disorders” (n = 39) are a included in the sample of adolescents with a psychiatric disorder in this table (n = 566), but were too diverse to be included in the three main groups of disorders (n = 451). The numbers in this table, for example 393/560 (70.4), indicate that 393 out of 560 adolescents with a psychiatric disorder had chronic pain, which show that we had six missing values (n = 566). This applies to the entire table.

In the total sample (n = 566) of adolescents with psychiatric disorders, girls reported a higher frequency of chronic pain than boys did (82.7% vs 55.1%, *P* < 0.001). Corresponding results were found among those in the MA-group, H-group and HMA-group. Girls also reported a higher frequency of chronic pain in all three locations (*P* < 0.05), as well as multisite pain (*P* < 0.001). Furthermore, in the MA-group and in the H-group, girls reported more pain-related disability (*P* < 0.05) than boys did.

Separate analyses stratified by sex showed that frequency of chronic pain (in all three locations), multisite pain and pain-related disability in girls was higher in the MA-group than in the H-group, and even higher in the HMA-group, yet not all differences were statistically significant. Girls in the MA-group experienced a higher frequency of chronic headache than girls in the H-group (63.0% vs 30.6%, *P* = 0.005), and they also reported more chronic abdominal pain (54.5% vs 19.6%, *P* = 0.003) and multisite pain (56.3% vs 26.9%, *P* = 0.032).

Girls in the HMA-group experienced a higher frequency of multisite pain than girls in in the MA-group did (80.0% vs 56.3%, *P* = 0.025), and compared with the H-group, they also reported a higher frequency of chronic headaches (68.0% vs 43.2%, *P* = 0.032), chronic abdominal pain (60.0% vs 33.3%, *P* = 0.018), and multisite pain (80.0% vs 41.3%, *P* = 0.001). For boys, the frequency of pain-related disability was significantly higher among boys in the HMA-group than those in the H-group (33.3% vs 12.4%, *P* = 0.032). The frequency of pain did not differ between boys in the MA-group and those in the H-group (see raw data in Table 2).

### Risk of chronic pain and pain-related disability

Adolescents in the MA-group had a higher risk of chronic pain (OR = 1.83, CI 1.19–2.83, *P* = 0.006) and pain-related disability (OR = 1.78, CI 1.12–2.85, *P* = 0.016) than those in the H-group did. Furthermore, adolescents in the HMA-group had an even higher increased risk of pain-related disability (OR = 2.35, CI 1.16–4.97, *P* = 0.025) than those in the H-group (see Table 3). However, when analyses were performed by sex, the associations above disappeared (data not shown).

**Table 3:** Logistic regression with chronic pain and pain-related disability as dependent variables, and with groups of disorders as independent variables

	**Risk of chronic pain**			**Risk of pain-related disability**		
**Overall**	n	OR (95% CI)	*P*	n	OR (95% CI)	*P*
*Unadjusted*	447			449		
H-group (ref)		1			1	
MA-group		1.83 (1.19 to 2.83)	0.006		1.78 (1.12 to 2.85)	0.016
HMA-group		1.89 (0.85 to 4.22)	0.118		2.35 (1.16 to 4.97)	0.025
*Adjusted separately for*						
**Sex**	447			449		
H-group (ref)		1			1	
MA-group		1.27 (0.80 to 2.03)	0.316		1.40 (0.86 to 2.29)	0.182
HMA-group		1.49 (0.65 to 3.43)	0.345		2.00 (0.93 to 4.29)	0.075
**Age**	447			449		
H-group (ref)		1			1	
MA-group		1.62 (1.04 to 2.52)	0.035		1.82 (1.13 to 2.93)	0.013
HMA-group		1.84 (0.82 to 4.13)	0.142		2.37 (1.12 to 5.01)	0.024
**Socioeconomic status**	314			316		
H-group (ref)		1			1	
MA-group		1.78 (1.06 to 2.98)	0.030		1.74 (0.98 to 3.07)	0.057
HMA-group		2.06 (0.83 to 5.10)	0.117		2.74 (1.19 to 6.31)	0.018
**Adjusted for all**	314			316		
H-group (ref)		1			1	
MA-group		1.14 (0.65 to 2.01)	0.643		1.40 (0.77 to 2.55)	0.274
HMA-group		1.65 (0.63 to 4.28)	0.306		2.35 (1.00 to 5.52)	0.051

H-group represents those with hyperkinetic disorders; MA-group represents mood or anxiety disorders; HMA-group represents hyperkinetic *and* mood or anxiety disorders.

Furthermore, the increased risk of chronic pain and pain-related disability in the MA-group was no longer significant after adjusting for sex. For adolescents in the HMA-group, although not significant, the risk of chronic pain increased when adjusted for SES (OR = 2.06, CI 0.83–5.10, *P* = 0.117). Furthermore, the increased risk of pain-related disability among adolescents in the HMA-group was still high when adjusted for sex (OR = 2.00, CI 1.12–4.29, *P* = 0.075), and the risk increased when adjusted for age (OR = 2.37, CI 1.12–5.01, *P* = 0.024) and SES (OR = 2.74, CI 1.19–6.31, *P* = 0.018) as well. With adjustment for sex, age and SES in the final model, there was no increased risk of chronic pain, and no significantly increased risk of pain-related disability for those in the MA-group, but there was a borderline significant risk of pain-related disability in the HMA-group group compared with the H-group (OR = 2.35, CI 1.00–5.52, *P* = 0.051).

## Discussion

In this cross-sectional study of adolescents with psychiatric disorders, two-thirds reported chronic pain. One in five experienced pain-related disability, and reports of pain increased with age. Girls reported a higher frequency of chronic pain and pain-related disability than boys did. Adolescents with mood or anxiety disorders had a significantly higher frequency of chronic pain and pain-related disability than those with hyperkinetic disorders; however, this association practically disappeared when adjusting for sex. Adolescents with hyperkinetic *and* mood or anxiety disorders had a two to three-fold increased risk (fully adjusted) of pain-related disability compared to those with hyperkinetic disorders alone.

In our study, 70% of adolescent psychiatric patients reported chronic pain, a higher frequency than that in the general adolescent population [1]. A Dutch longitudinal study found that children continued to suffer from chronic pain and psychiatric disorders in adolescence and young adulthood [18]. In a literature review, Bair et al. found evidence of chronic pain in a mean 65% of adult patients with depression [3], while in our study the prevalence was 79% for the group with mood disorders, of which depression was the most prevalent disorder. Possible explanations for this high frequency of pain may be that somatization or chronic pain might act as a manifestation of a psychiatric disorder, or that pain is a specific symptom of the disorder [3]. Depression and chronic pain also have many common features, including fatigue, sleep disturbances, and deficits in memory and attention [3,19]. This is, possibly, explained by shared genetic factors, biological pathways and neurotransmitters, often labeled the depression–pain dyad [20,21]. Depression is also associated with pain in multiple areas of the body [22], and in our study, the mood disorder group had the highest frequency of multisite pain. Those in the MA-group reported a higher frequency of multisite pain than those in the H-group did.

Among adolescents with anxiety disorders in our study, three out of four reported chronic pain. Possible reasons for the high frequency may be the mutual maintenance model [23,24], which holds that components of anxiety disorders maintain or worsen symptoms of pain, and components of pain maintain or worsen the symptoms of anxiety disorders. In addition, the shared vulnerability model suggests that individual, possibly genetically influenced, factors predispose the development of anxiety and chronic pain [25]. Anxiety and depression often coincide [26], which may increase the risk of pain, and this could possibly explain the high frequencies of chronic pain in adolescents with anxiety and depression in our study.

Chronic musculoskeletal pain was the most frequent type of chronic pain reported by adolescents in this study. In some previous studies of adolescents in the general population, headaches were reported to have the highest prevalence, followed by abdominal pain and musculoskeletal pain [7,27]. Our results thereby extend previous findings, but the various definitions of musculoskeletal pain may explain these different results. A Spanish cross-sectional study of almost 30,000 adolescents and adults showed that neck and low back pain were associated with depression [28]. This is in keeping with our results, because those with mood disorders had the highest frequency of chronic musculoskeletal pain.

Adolescents in the MA-group reported a significantly higher frequency of chronic headaches, chronic musculoskeletal pain, and chronic abdominal pain than those in the H-group did. This is in line with previous studies, which found that anxiety and depression in adolescents are associated with headaches [29], chronic musculoskeletal pain [30] and abdominal pain [31]. The association between depression, anxiety, and headaches/migraines is proposed to be a result of a neurolimbic dysfunction [32], where shared pathways in the limbic system are altered. Limbic dysfunction, in addition to hypothalamic dysfunction, might also be a possible reason for headaches in adolescents with eating disorders [33], where abnormal levels of several neurotransmitters (e.g., dopamine) are present. Adolescents with eating disorders reported a high frequency of abdominal pain, which is in accordance with previous studies [34,35]. The abdominal pain in adolescents with eating disorders may be a direct consequence of their illness. Dysfunction of the digestive organs may arise because of weakened stomach muscles and possible damage to the nerves involved in digesting food [36].

Chronic pain is known to affect daily functioning greatly [37], and may worsen the quality of lives of sufferers compared with those without chronic pain [38]. In our study, pain-related disability in daily living was experienced by one in five adolescents, a finding that is supported by several other studies [39-41]. In a prospective longitudinal study, Hall et al. found that symptoms of depression may mediate the effect of pain on disability [42]. This may explain our findings of the high frequency of pain-related disability among adolescents with mood disorders. The management of chronic pain requires identification of the biological, psychological, and social aspects of the pain, as well as an understanding how these components interact [43].

We found that the frequency of chronic pain and multisite pain increased with age, which is in agreement with current evidence [7,27]. In our study, age had no effect on pain-related disability, which contradicts a previous German cross-sectional study with 749 adolescents and children reporting an increase in pain-related disability with age [41]. This may be explained by the use of different questions regarding the severity of pain-related disability and suggests that further studies should be conducted.

Chronic pain was reported significantly more frequently by girls than by boys, regardless of the location of pain. This is consistent with previous findings [7,10,12,27,41]. Girls reported a higher frequency of multisite pain, and this is also in line with other studies [27,44-46]. It is suggested that sex hormones influence pain thresholds and tolerance, making girls more sensitive to pain than boys [47], but such findings are inconsistent [10]. Males may also have a stronger analgesic response in the μ-opioid receptor in the brain than females have when experiencing pain [47], but the findings are ambiguous [48,49]. In addition to this biological mechanism, sex differences in pain perception are also thought to be mediated by psychological and sociocultural factors [47,50]. This may help us understand why girls generally report a higher frequency of pain than boys do. Girls also reported a higher frequency of pain-related disability than boys, which is in line with studies showing that, in the general population, girls report more restrictions on daily living due to pain than boys do [41,51].

In some studies, low SES is reported to be associated with more chronic pain in adolescents, but the findings are conflicting [7]. SES is often measured as a combination of education, income, and occupation. In this study we used only parents’ education as proxy for SES, which may not be comprehensive enough to provide the differentiated information needed in the adolescents’ SES. Still, the difference in chronic pain in the MA-group versus the H-group could not be explained by education as proxy for SES, but rather it was explained by the high frequency of pain, as well as the presence of mood or anxiety disorders in girls.

A study of adolescents and adults in the general population showed that pain-related disability was associated with low SES [52]. In our study, the increased risk of pain-related disability in adolescents in the HMA-group compared with those in the H-group is in agreement with previous findings, showing that the presence of multiple psychiatric disorders was associated with greater pain-related disability in adults and adolescents, compared with that of individuals with one disorder [2].

Our study had several strengths; it included a relatively large clinical sample, providing a high degree of precision in the estimates. Psychiatric diagnoses were classified by a clinician according to the ICD-10, thereby removing the limitations of using self-report measures to establish psychopathology. Unlike previous research, we investigated chronic pain across diagnoses to discover associations between psychiatric diagnostic groups and chronic pain and pain-related disability.

Some limitations of this study need to be taken into account. Girls were somewhat overrepresented in the study group compared with the non-participating group, which may contribute to the high frequency of pain in the total sample. However, we adjusted for sex in the risk analyses, and sex did not explain the increased risk of pain-related disability among adolescents in the HMA-group.

The participants were representative with regard to reasons for referral, coded according to a national classification system of suspected disorders. Although the reason for referral indicates the main problem area that will be subjected to examination, it may not coincide completely with the final diagnosis. Hence, our results may not be applicable to other populations and study settings. Inter-rater reliability for diagnostic assessment was not available. However diagnoses were made by an experienced child psychiatrist or psychologist after arriving at a consensus with professionals from the multi-disciplinary team adhering to national guidelines and procedures.

We measured subjective pain complaints among adolescents using self-report questionnaires. The adolescents reported their pain complaints both currently and retrospectively for the three months prior to assessment. Retrospective reports may be influenced by recall bias, which in turn could have led to under- or overestimation of pain in this study. During the study period, 2032 adolescent patients visited the CAP clinic at least once. Of these, 95 were missing registration data, and hence, were not included in study recruitment. Furthermore, only 43% of the eligible and invited patients participated in the CAP survey, which means that this study requires replication. Because a control group was not included in the study design, the results were discussed in relation to the extensive literature on this subject.

## Conclusions

In this study, seven out of 10 adolescents with psychiatric disorders reported chronic pain. Furthermore, pain generally increased with age, and girls experienced more chronic pain than boys did. Adolescents with mood or anxiety disorders had a higher risk of chronic pain than adolescents with hyperkinetic disorders did; however, this could be explained by sex. Comorbidity between hyperkinetic *and* mood or anxiety disorders entailed a twofold increased risk of pain-related disability. The underpinning mechanisms behind these relationships involve biological, psychological and sociocultural factors. For clinicians, awareness and early detection of chronic pain in adolescents with psychiatric disorders is important. Both conditions emerge early in life and are associated with poor quality of life and poor long-term outcomes. Nevertheless, these conditions are potentially modifiable and treatable, yet adequate treatment depends on diagnosing both correctly. Clinicians should routinely include an evaluation of pain and relevant somatic aspects in the clinical assessment. Similarly, clinicians who treat pain should include a short psychiatric screening in their assessment. Further research should supplement present knowledge by using a prospective design to investigate both the causal relationships and long-term course and outcome of comorbid pain and psychiatric disorders. Future studies should also evaluate the effect of interventions or treatment that aim to reduce long-term disability.

## Competing interests

The authors declare that there are no financial or other relationships that might lead to a conflict of interest.

## Authors’ contributions

WLM designed and drafted the manuscript with guidance from MSI and OB. WLM conducted the analysis and interpreted the data with guidance from SL. MSI, OB, and SL revised the manuscript for important intellectual content. All authors gave final approval on the version to be published.

## Pre-publication history

The pre-publication history for this paper can be accessed here:

http://www.biomedcentral.com/1471-244X/13/272/prepub

## References

[B1] Mykletun A Knudsen AK Mathiesen KS Psykiske lidelser i norge: Et folkehelseperspektiv. [Mental disorders in norway: a public health perspective] 2009 Oslo: Norwegian Institute of Public Health

[B2] McWilliams LA Cox BJ Enns MW Mood and anxiety disorders associated with chronic pain: an examination in a nationally representative sample Pain 2003 106 127 133 10.1016/S0304-3959(03)00301-4 14581119

[B3] Bair MJ Robinson RL Katon W Kroenke K Depression and pain comorbidity: a literature review Arch Intern Med 2003 163 2433 2445 10.1001/archinte.163.20.2433 14609780

[B4] Means-Christensen AJ Roy-Byrne PP Sherbourne CD Craske MG Stein MB Relationships among pain, anxiety, and depression in primary care Depress Anxiety 2008 25 593 600 10.1002/da.20342 17932958

[B5] Katona C Peveler R Dowrick C Wessely S Feinmann C Gask L Lloyd H Williams AC Wager E Pain symptoms in depression: definition and clinical significance Clin Med 2005 5 390 395 10.7861/clinmedicine.5-4-390 16138496 PMC4954214

[B6] Norwegian Institute of Public Health Facts About Chronic Pain Prevalence [http://www.fhi.no/eway/default.aspx?pid=240&trg=List_6673&Main_6664=6894:0:25,7583:1:0:0:::0:0&MainContent_6894=6671:0:25,7590:1:0:0:::0:0&List_6673=6674:0:25,7602:1:0:0:::0:0]

[B7] King S Chambers CT Huguet A MacNevin RC McGrath PJ Parker L MacDonald AJ The epidemiology of chronic pain in children and adolescents revisited: a systematic review Pain 2011 152 2729 2738 10.1016/j.pain.2011.07.016 22078064

[B8] Rustoen T Wahl AK Hanestad BR Lerdal A Paul S Miaskowski C Prevalence and characteristics of chronic pain in the general Norwegian population Eur J Pain 2004 8 555 565 10.1016/j.ejpain.2004.02.002 15531224

[B9] Rustoen T Wahl AK Hanestad BR Lerdal A Paul S Miaskowski C Gender differences in chronic pain–findings from a population-based study of Norwegian adults Pain Manag Nurs 2004 5 105 117 10.1016/j.pmn.2004.01.004 15359222

[B10] Fillingim RB King CD Ribeiro-Dasilva MC Rahim-Williams B Riley JL 3rd Sex, gender, and pain: a review of recent clinical and experimental findings J Pain 2009 10 447 485 19411059 10.1016/j.jpain.2008.12.001 PMC2677686

[B11] Nicolson SE Caplan JP Williams DE Stern TA Comorbid pain, depression, and anxiety: multifaceted pathology allows for multifaceted treatment Harv Rev Psychiatry 2009 17 407 420 10.3109/10673220903463226 19968455

[B12] Hoftun GB Romundstad PR Zwart JA Rygg M Chronic idiopathic pain in adolescence–high prevalence and disability: the young HUNT study 2008 Pain 2011 152 2259 2266 10.1016/j.pain.2011.05.007 21683528

[B13] Campo JV Comer DM Jansen-Mcwilliams L Gardner W Kelleher KJ Recurrent pain, emotional distress, and health service use in childhood J Pediatr 2002 141 76 83 10.1067/mpd.2002.125491 12091855

[B14] DeSocio J Hootman J Children’s mental health and school success J Sch Nurs 2004 20 189 196 10.1177/10598405040200040201 15283617

[B15] WHO The ICD-10 Classification of Mental and Behavioural Disorders, Clinical Description and Diagnostic Guidelines 1992 Geneva: World Health Organization

[B16] Mikkelsson M Salminen JJ Kautiainen H Non-specific musculoskeletal pain in preadolescents. Prevalence and 1-year persistence Pain 1997 73 29 35 10.1016/S0304-3959(97)00073-0 9414054

[B17] Mikkelsson M Salminen JJ Sourander A Kautiainen H Contributing factors to the persistence of musculoskeletal pain in preadolescents: a prospective 1-year follow-up study Pain 1998 77 67 72 10.1016/S0304-3959(98)00083-9 9755020

[B18] Knook LM Lijmer JG Konijnenberg AY Taminiau B van Engeland H The course of chronic pain with and without psychiatric disorders: a 6-year follow-up study from childhood to adolescence and young adulthood J Clin Psychiatry 2012 73 e134 e139 10.4088/JCP.10m06751 22316584

[B19] Sharp J Keefe B Psychiatry in chronic pain: a review and update Curr Psychiatry Rep 2005 7 213 219 10.1007/s11920-005-0056-x 15935136

[B20] Lindsay PG Wyckoff M The depression-pain syndrome and its response to antidepressants Psychosomatics 1981 22 571–573 576–577 7267947 10.1016/S0033-3182(81)73478-9

[B21] Goldenberg DL Pain/depression dyad: a key to a better understanding and treatment of functional somatic syndromes Am J Med 2010 123 675 682 10.1016/j.amjmed.2010.01.014 20541169

[B22] Gambassi G Pain and depression: the egg and the chicken story revisited Arch Gerontol Geriatr 2009 49 Suppl 1 103 112 19836622 10.1016/j.archger.2009.09.018

[B23] Asmundson GJ Katz J Understanding the co-occurrence of anxiety disorders and chronic pain: state-of-the-art Depress Anxiety 2009 26 888 901 10.1002/da.20600 19691031

[B24] Sharp TJ Harvey AG Chronic pain and posttraumatic stress disorder: mutual maintenance? Clin Psychol Rev 2001 21 857 877 10.1016/S0272-7358(00)00071-4 11497210

[B25] Asmundson GJ Coons MJ Taylor S Katz J PTSD and the experience of pain: research and clinical implications of shared vulnerability and mutual maintenance models Can J Psychiatry 2002 47 930 937 12553128 10.1177/070674370204701004

[B26] Andreescu C Lenze EJ Comorbid anxiety and depression: bete noire or quick fix? Br J Psychiatry 2012 200 179 181 10.1192/bjp.bp.111.097022 22383763 PMC3619974

[B27] Perquin CW Hazebroek-Kampschreur AA Hunfeld JA Bohnen AM van Suijlekom-Smit LW Passchier J van der Wouden JC Pain in children and adolescents: a common experience Pain 2000 87 51 58 10.1016/S0304-3959(00)00269-4 10863045

[B28] Fernandez-de-las-Penas C Hernandez-Barrera V Alonso-Blanco C Palacios-Cena D Carrasco-Garrido P Jimenez-Sanchez S Jimenez-Garcia R Prevalence of neck and low back pain in community-dwelling adults in Spain: a population-based national study Spine (Phila Pa 1976) 2011 36 E213 E219 10.1097/BRS.0b013e3181d952c2 21079541

[B29] Antonaci F Nappi G Galli F Manzoni GC Calabresi P Costa A Migraine and psychiatric comorbidity: a review of clinical findings J Headache Pain 2011 12 115 125 10.1007/s10194-010-0282-4 21210177 PMC3072482

[B30] O’Sullivan P Beales D Jensen L Murray K Myers T Characteristics of chronic non-specific musculoskeletal pain in children and adolescents attending a rheumatology outpatients clinic: a cross-sectional study Pediatr Rheumatol Online J 2011 9 3 10.1186/1546-0096-9-3 21247439 PMC3034682

[B31] Di Lorenzo C Colletti RB Lehmann HP Boyle JT Gerson WT Hyams JS Squires RH Jr Walker LS Kanda PT Subcommittee AAP Pain NCoCA Chronic abdominal pain in children: a technical report of the American academy of pediatrics and the North American society for pediatric gastroenterology, hepatology and nutrition J Pediatr Gastroenterol Nutr 2005 40 249 261 10.1097/01.MPG.0000154661.39488.AC 15735476

[B32] Maizels M Aurora S Heinricher M Beyond neurovascular: migraine as a dysfunctional neurolimbic pain network Headache 2012 52 1553 1565 10.1111/j.1526-4610.2012.02209.x 22757613

[B33] D’Andrea G Ostuzzi R Francesconi F Musco F Bolner A D’Onofrio F Colavito D Migraine prevalence in eating disorders and pathophysiological correlations Neurol Sci 2009 30 1 S55 S59 10.1007/s10072-008-0003-9 19415427

[B34] Dorn LD Campo JC Thato S Dahl RE Lewin D Chandra R Di Lorenzo C Psychological comorbidity and stress reactivity in children and adolescents with recurrent abdominal pain and anxiety disorders J Am Acad Child Adolesc Psychiatry 2003 42 66 75 10.1097/00004583-200301000-00012 12500078

[B35] Dufton LM Dunn MJ Compas BE Anxiety and somatic complaints in children with recurrent abdominal pain and anxiety disorders J Pediatr Psychol 2009 34 176 186 18577541 10.1093/jpepsy/jsn064 PMC2645470

[B36] Weiselberg EC Gonzalez M Fisher M Eating disorders in the twenty-first century Minerva Ginecol 2011 63 531 545 22036757

[B37] Palermo TM Assessment of chronic pain in children: current status and emerging topics Pain Res Manag 2009 14 21 26 19262912 10.1155/2009/236426 PMC2706560

[B38] Huguet A Miro J The severity of chronic pediatric pain: an epidemiological study J Pain 2008 9 226 236 10.1016/j.jpain.2007.10.015 18088558

[B39] Konijnenberg AY Uiterwaal CS Kimpen JL van der Hoeven J Buitelaar JK de Graeff-Meeder ER Children with unexplained chronic pain: substantial impairment in everyday life Arch Dis Child 2005 90 680 686 10.1136/adc.2004.056820 15899922 PMC1720481

[B40] Petersen S Hagglof BL Bergstrom EI Impaired health-related quality of life in children with recurrent pain Pediatrics 2009 124 e759 e767 10.1542/peds.2008-1546 19736269

[B41] Roth-Isigkeit A Thyen U Stoven H Schwarzenberger J Schmucker P Pain among children and adolescents: restrictions in daily living and triggering factors Pediatrics 2005 115 e152 e162 10.1542/peds.2004-0682 15687423

[B42] Hall AM Kamper SJ Maher CG Latimer J Ferreira ML Nicholas MK Symptoms of depression and stress mediate the effect of pain on disability Pain 2011 152 1044 1051 10.1016/j.pain.2011.01.014 21306826

[B43] Gatchel RJ Peng YB Peters ML Fuchs PN Turk DC The biopsychosocial approach to chronic pain: scientific advances and future directions Psychol Bull 2007 133 581 624 17592957 10.1037/0033-2909.133.4.581

[B44] Petersen S Brulin C Bergstrom E Recurrent pain symptoms in young schoolchildren are often multiple Pain 2006 121 145 150 10.1016/j.pain.2005.12.017 16473464

[B45] Smedbraten BK Natvig B Rutle O Bruusgaard D Self-reported bodily pain in schoolchildren Scand J Rheumatol 1998 27 273 276 9751467

[B46] Hoftun GB Romundstad PR Rygg M Factors associated with adolescent chronic non-specific pain, chronic multisite pain, and chronic pain with high disability: the Young-HUNT Study 2008 J Pain 2012 13 874 883 10.1016/j.jpain.2012.06.001 22832694

[B47] Wiesenfeld-Hallin Z Sex differences in pain perception Gend Med 2005 2 137 145 10.1016/S1550-8579(05)80042-7 16290886

[B48] Racine M Tousignant-Laflamme Y Kloda LA Dion D Dupuis G Choiniere M A systematic literature review of 10 years of research on sex/gender and pain perception - part 2: do biopsychosocial factors alter pain sensitivity differently in women and men? Pain 2012 153 619 635 10.1016/j.pain.2011.11.026 22236999

[B49] Mogil JS Bailey AL Sex and gender differences in pain and analgesia Prog Brain Res 2010 186 141 157 21094890 10.1016/B978-0-444-53630-3.00009-9

[B50] Smitherman TA Ward TN Psychosocial factors of relevance to sex and gender studies in headache Headache 2011 51 923 931 10.1111/j.1526-4610.2011.01919.x 21631477

[B51] Unruh AM Gender variations in clinical pain experience Pain 1996 65 123 167 10.1016/0304-3959(95)00214-6 8826503

[B52] Dorner TE Muckenhuber J Stronegger WJ Rasky E Gustorff B Freidl W The impact of socio-economic status on pain and the perception of disability due to pain Eur J Pain 2011 15 103 109 10.1016/j.ejpain.2010.05.013 20558096

